# Antibody Persistence in Adults Two Years after Vaccination with an H1N1 2009 Pandemic Influenza Virus-Like Particle Vaccine

**DOI:** 10.1371/journal.pone.0150146

**Published:** 2016-02-26

**Authors:** Nuriban Valero-Pacheco, Marisol Pérez-Toledo, Miguel Ángel Villasís-Keever, Adriana Núñez-Valencia, Ilka Boscó-Gárate, Bernardo Lozano-Dubernard, Horacio Lara-Puente, Clara Espitia, Celia Alpuche-Aranda, Laura C. Bonifaz, Lourdes Arriaga-Pizano, Rodolfo Pastelin-Palacios, Armando Isibasi, Constantino López-Macías

**Affiliations:** 1 Medical Research Unit on Immunochemistry, Specialties Hospital, National Medical Centre “Siglo XXI”, Mexican Social Security Institute, Mexico City, D.F., Mexico; 2 Departamento de Inmunología, Escuela Nacional de Ciencias Biológicas, Instituto Politécnico Nacional, México, D.F., México; 3 Unidad de Investigación en Epidemiología Clínica, Hospital de Pediatría, Centro Médico Nacional “Siglo XXI”, Instituto Mexicano del Seguro Social, México, D.F., México; 4 Departamento de Investigación y Desarrollo, Laboratorio Avi-Mex, S.A. de C.V., México, D.F., México; 5 Departamento de Inmunología, Instituto de Investigaciones Biomédicas, Universidad Nacional Autónoma de México, México, D.F., México; 6 Instituto Nacional de Salud Pública, Cuernavaca, Morelos, México; 7 Facultad de Química, Universidad Nacional Autónoma de México, México, D.F., México; 8 Nuffield Department of Medicine, University of Oxford, Oxford, United Kingdom; Icahn School of Medicine at Mount Sinai, UNITED STATES

## Abstract

The influenza virus is a human pathogen that causes epidemics every year, as well as potential pandemic outbreaks, as occurred in 2009. Vaccination has proven to be sufficient in the prevention and containment of viral spreading. In addition to the current egg-based vaccines, new and promising vaccine platforms, such as cell culture-derived vaccines that include virus-like particles (VLPs), have been developed. VLPs have been shown to be both safe and immunogenic against influenza infections. Although antibody persistence has been studied in traditional egg-based influenza vaccines, studies on antibody response durations induced by VLP influenza vaccines in humans are scarce. Here, we show that subjects vaccinated with an insect cell-derived VLP vaccine, in the midst of the 2009 H1N1 influenza pandemic outbreak in Mexico City, showed antibody persistence up to 24 months post-vaccination. Additionally, we found that subjects that reported being revaccinated with a subsequent inactivated influenza virus vaccine showed higher antibody titres to the pandemic influenza virus than those who were not revaccinated. These findings provide insights into the duration of the antibody responses elicited by an insect cell-derived pandemic influenza VLP vaccine and the possible effects of subsequent influenza vaccination on antibody persistence induced by this VLP vaccine in humans.

## Introduction

The influenza virus is a human pathogen that causes epidemics every year, as well as potential pandemic outbreaks, as occurred in 2009 [1]. Conventional influenza vaccines are based on purified and inactivated egg-grown virus. However, during the 2009 influenza pandemic, the worldwide capacity for producing traditional influenza vaccines failed to provide sufficient vaccine dosages within a timely manner. Alternatively, novel approaches including mammal, plant, and insect cell culture-derived technologies have been used to produce whole-virus, subunits or virus-like particle (VLP) vaccines [2].

VLPs are nanoparticles composed of a non-infectious subset of viral components that mimic the wild-type virus structure but lack viral genetic material [3]. Therefore, they represent a repetitive high-density display of viral surface antigens, which make them a promising vaccine platform. Additionally, VLPs can also be exploited as scaffolds to express heterogeneous molecular arrays of viral antigens, including hepatitis B (HBV) capsid, human papillomavirus (HPV), hepatitis E virus (HEV), Norwalk virus, and influenza virus amongst others [4]. Furthermore, VLP-based vaccines that targeted various pathogens were shown to be both safe and immunogenic in clinical trials [4–16]. Currently, two VLP-based vaccines (HBV and HPV) are already approved for human use [4]. Specifically, the VLP-based HPV vaccine was proven to induce persistent antibody titres for up to four to six years after vaccination [17, 18].

Antibody persistence elicited by egg-based seasonal influenza vaccines has been widely studied. Clinical trials in children, adults, and the elderly found that significant antibody titres induced by traditional vaccination to the influenza virus were detectable up to 18 months post-vaccination [19–29]. In line with these findings, clinical trials analysing long-term antibody responses to H5N1 avian influenza vaccines also found similar results [30–35]. Likewise, several studies addressing long-term antibody responses that were induced by egg-grown 2009 pandemic influenza A (H1N1) virus vaccines [A(H1N1)pdm09] in different populations (children, the elderly, and adults) reported vaccine-induced antibody persistence that was detectable up to 12 months after vaccination [36–46]. However, clinical trials evaluating the antibody response durations that are induced by VLP-based influenza vaccines are scarce [12].

In this study, we found that after 24 months of VLP vaccination, antibody titres were higher in subjects that were vaccinated with an insect cell-derived H1N1 2009 pandemic influenza VLP vaccine, compared with a placebo. Moreover, we observed that subjects who reported being revaccinated with a trivalent inactivated influenza virus vaccine (IIV), showed higher antibody titres to the A(H1N1)pdm09 virus in comparison with those who did not receive IIV. These findings provide insights into the duration of the antibody responses that are elicited by an insect cell-derived pandemic influenza VLP vaccine and the possible effects of subsequent influenza vaccination on antibody persistence induced by this VLP vaccine in humans.

## Results

### Study demographics

This is a cross-sectional study from a cohort of subjects who had previously participated in a phase 2, randomised, double blind, placebo-controlled study to evaluate the safety, tolerability, and immunogenicity of an experimental and unlicensed influenza VLP vaccine that was produced by Novavax against the pandemic A/California/04/2009 H1N1 virus (ClinicalTrials.gov Identifier: NCT01072799) [14]. The preceding study was carried out in two stages (S1 Fig). Part A was performed to evaluate the safety and immunogenicity of three doses of VLP vaccine. In this part subjects were immunised with either 5 μg, 15 μg or 45 μg of a haemagglutinin (HA) VLP vaccine or a placebo, with a boost on day 22. Blood samples were taken at day 1, 14, and 36. Whereas in Part B of the previous study, the volunteers received a single dose of 15 μg VLP or placebo injection on day 1 and assessed for safety. No blood samples were taken from Part B subjects.

For the current study, a representative sample comprising subjects from Part A and Part B from the previous VLP study were recruited (n = 489). The median age was 37.0 years old with a range of 19–65 years old. From Part A, 263 subjects were enrolled, including 75 individuals from the 5 μg haemagglutinin (HA) group, 86 persons from the 15 μg HA group, 72 subjects from the 45 μg HA group, and 30 individuals who received the placebo. From Part B, 226 subjects were enrolled, including 165 subjects who received 15 μg of VLP vaccine and 61 individuals who received placebo. Enrolment and sample collection of all of the subjects analysed in the study occurred at a median period of 24 months (range from 17–29 months) post first VLP vaccination. In the case of Part A (two VLP doses), the sample collection and antibody persistence analysis were performed at 25 months (range from 22–29 months) after VLP vaccination; conversely, the samples from Part B (one VLP dose) were obtained 21 months (range from 17–24 months) post vaccination. All the groups showed comparable age and gender distributions with each other, as shown in Table 1.

**Table 1:** Study population demographics and characteristics.

	Parts A+B	Part A. Two doses					Part B. One dose		
	Total (489)	Total (263)	5 μg (75)	15 μg (86)	45 μg (72)	Placebo (30)	Total (226)	15 μg (165)	Placebo (61)
**Women (%)**	306 (62.6)	150 (57)	36 (48)	57 (66)	39 (54)	18 (60)	156 (69)	113 (68)	43 (70)
**Men (%)**	183 (37.4)	113 (43)	39 (52)	29 (34)	33 (46)	12 (40)	70 (31)	52 (32)	18 (30)
**Median age (years old, range)**	37.0 (19–65)	35.0 (20–65)	35.0 (20–64)	34.5 (20–65)	38.5 (20–65)	28.5 (22–65)	39.0 (19–64)	40.0 (19–64)	36.0 (19–63)
**Months after VLP (median, range)**	24.0 (17–29)	25.0 (22–29)	25.0 (22–29)	25.0 (22–29)	25.0 (22–28)	25.0 (22–26)	21.0 (17–24)	21.0 (17–24)	20.0 (17–24)
**Influenza-like Illness (ILI, %)**	94 (19.2)	51 (19.4)	14 (18.7)	17 (19.8)	16 (22.2)	4 (13.3)	43 (19.0)	32 (19.4)	11 (18.0)
**IIV vaccination after VLP (%)**	100 (20.4)	54 (20.5)	15 (20.0)	22 (25.6)	12 (16.7)	5 (16.7)	46 (20.4)	34 (20.6)	12 (19.7)

IIV: inactivated influenza virus vaccine.

Ninety-four of the subjects (19.2%) reported having at least one episode of an influenza-like illness (ILI), which was defined as a measured fever ≥ 38°C and a cough with onset occurring within the last 10 days [47]; however, no differences were found in the ILI frequency between the Part A and B groups (19.4% and 19.0%, respectively) (Table 1). Additionally, none of the recruited subjects reported having been diagnosed with an influenza virus infection between the VLP vaccination and blood sampling 24 months later. One hundred subjects (20.4%) of all the volunteers analysed reported having been vaccinated with IIV after immunisation with the VLP vaccine; however, volunteers from parts A and B showed a comparable proportion of individuals who were vaccinated with IIV (20.5% and 20.4%, respectively) (Table 1). The antibody responses of these volunteers were analysed as a separate group from the non-IIV revaccinated subjects.

### Antibodies to the A(H1N1)pdm09 virus can still be detected up to 25 months after the VLP vaccination

Next, we questioned if antibody levels could still be detectable in VLP recipient subjects after approximately 24 months; hence, we measured A(H1N1)pdm09 virus antibody titres with an haemagglutination inhibition assay (HI). In the Part A subjects, from whom the samples were taken after 25 months of VLP immunisation, we found that irrespective of the VLP dose, the vaccinated subjects showed similar antibody titres (GMT 13.21) compared with those who received the placebo (GMT 10.16) (Table 2). Furthermore, we found that 52.2% of the VLP vaccinated subjects had detectable antibody titres (>1:10), while 22.8% had seroprotective antibody levels (>1:40). However, these antibody levels were not different from those found in the placebo group (44% and 20%, respectively) (Table 3).

**Table 2:** Persistence of haemagglutination inhibition (HI) antibody responses to the A(H1N1)pdm09 influenza virus in the non-IIV revaccinated subjects.

	VLP Dose	No. subjects	GMT	95% CI	*P* value*
**Part A–Two VLP doses (25 months p.i.)**	5 μg HA	60	12.56	9.53–17.26	0.38
	15 μg HA	64	12.65	9.29–17.43	0.51
	45 μg HA	60	14.54	10.77–19.41	0.17
	VLP	184	13.21	10.93–15.80	0.29
	Placebo	25	10.16	8.95–52.46	---
**Part B–One VLP dose (21 months p.i.)**	15 μg HA	131	15.14	2.44–18.76	**0.002**
	Placebo	49	8.55	6.83–10.86	

*P value respective to each placebo group.

**Table 3:** Persistence of seroprevalent and seroprotective antibody responses to the A(H1N1)pdm09 influenza virus in both the non-IIV and IIV revaccinated subjects.

	Dose	No. subjects	≥1:10 (%)	*P* value*	≥ 1:40 (%)	*P* value*
**VLP**						
Part A	VLP	184	52.2	0.44	22.8	0.75
	Placebo	25	44.0		20.0	
Part B	15 μg HA	131	57.3	**0.003**	29.8	**0.004**
	Placebo	49	32.7		10.2	
**VLP + IIV**						
Part A	VLP	49	79.6	0.73	57.1	0.13
	Placebo	5	80.0		20.0	
Part B	15 μg HA	34	73.5	0.45	41.2	0.26
	Placebo	12	66.7		25.0	

*P value respective to each Placebo group. IIV: inactivated influenza virus vaccine.

Conversely, in the Part B subjects, from whom the samples were taken 21 months after VLP vaccination, we found that the vaccinated subjects had higher antibody titres (GMT 15.14) than the placebo recipients (GMT 8.55) (*P* = 0.002, Table 2). Additionally, we found that 57.3% of the VLP vaccinated subjects still showed detectable antibody titres, while 29.8% had seroprotective antibody levels. Moreover, the seroprevalence (*P* = 0.003) and seroprotection levels (*P* = 0.004) were both significantly higher in the VLP vaccinated subjects than in the placebo recipients (32.7% and 10.2%, respectively) (Table 3). In addition, when the 15μg VLP arms from both Part A and B were combined and compared to the placebo recipients, we found that VLP vaccinated subjects had higher antibody titres (GMT 13.97, 95% CI 11.89–16.57) compared to the placebo group (GMT 7.86, 95% CI 6.60–9.44) (*P*<0.001),

### Seasonal inactivated influenza virus vaccination boosted VLP induced antibody titres to the A(H1N1)pdm09 virus

**Fig 1:**
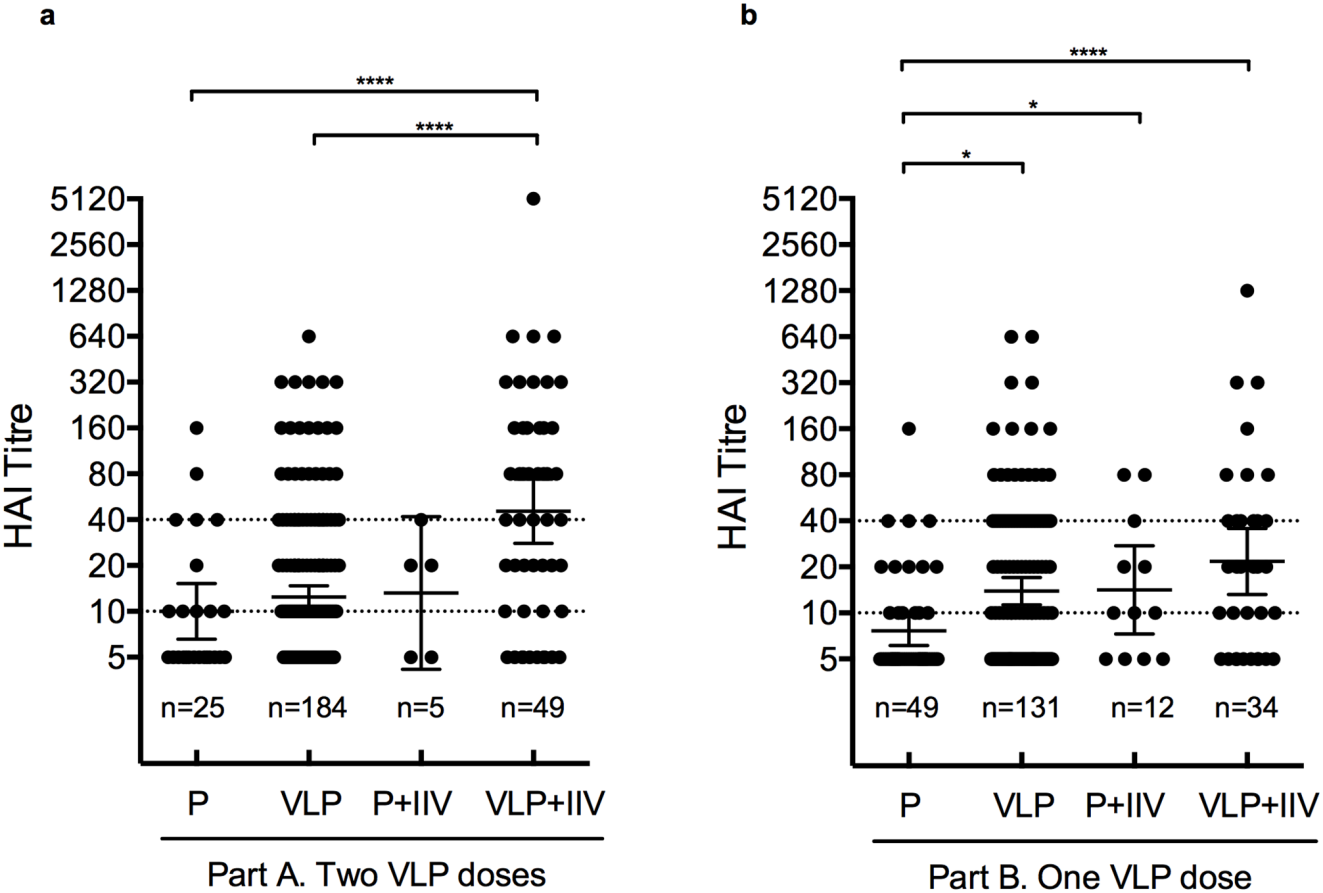
Seasonal trivalent inactivated vaccination boosted VLP-induced antibody titres to A(H1N1)pdm09 virus. Haemagglutination inhibition (HI) titres from the VLP or placebo recipients (P) who did or did not receive seasonal inactivated vaccine (IIV) after VLP were evaluated in the Part A (a) and Part B subjects (b). Scatter dot plots with GMT with 95% CI for the HI titres are shown for each group. The antibody titres were analysed with the nonparametric 1-way ANOVA (Kruskal-Wallis test) and with the U-Mann-Whitney post-hoc multiple comparisons test (*P<0.05, ****P<0.0001).

Since 20.4% (n = 100) of the volunteers reported receiving IIV in the time between VLP vaccination and blood sample collection for this study, we questioned if revaccination with IIV would increase antibody titres that were induced by VLP vaccination. We found that the Part A subjects who reported receiving IIV after VLP showed higher antibody titres than the VLP (*P*<0.0001) and placebo recipients (*P*<0.001) who were not revaccinated with IIV (Fig 1a). In the Part B subjects, we found that VLP (*P*<0.001) and placebo recipients (*P*<0.05) who reported receiving IIV had higher antibody titres when compared with the placebo treated subjects without IIV revaccination (Fig 1b).

The seroprevalence frequency of the antibody levels (>1:10) amongst the VLP subjects who reported receiving IIV was higher (Part A, 79.6% and Part B, 73.5%), compared with the non-IIV revaccinated VLP subjects (Part A, 52.2% and Part B, 57.3%). Likewise, the seroprotection frequency (>1:40) amongst the VLP subjects who reported receiving IIV was higher (Part A, 57.1% and Part B, 41.2%) compared with the non-IIV revaccinated VLP subjects (Part A, 22.8% and Part B, 29.8%). No differences between the VLP and placebo groups were found in the groups that reported receiving IIV (VLP or placebo) from either the Part A or B subjects (Table 3).

## Discussion

The duration of antibody responses that are induced by egg-produced influenza vaccines have been studied thoroughly. These reports include vaccines against seasonal [19–29], avian H5N1 [30–35], and pandemic H1N1 viruses [39–46, 48–51]. These studies showed that antibody persistence was found up to 12 months after vaccination with the avian and pandemic vaccines, and up to 18 months for seasonal influenza vaccines. These studies have helped to improve immunisation programmes; however, in the case of VLP-based vaccines, these schemes have not yet been explored in humans.

Although several VLP-based vaccines candidates against seasonal, H5N1, and pandemic H1N1 influenza viruses have been developed, only some of these candidates have been tested in clinical trials, showing to be both safe and immunogenic [11–16]. However, to date, only a single report on the duration of antibody responses that are induced by influenza VLP vaccines in humans has been published [12].

In a previous Phase II clinical trial that was conducted in the midst of a pandemic in Mexico, a non-adjuvanted monovalent insect cell-derived H1N1 2009 pandemic influenza VLP vaccine candidate was found to be both immunogenic and well tolerated in healthy adults [14]. In the current study, the persistence of antibody responses that were induced by this VLP vaccine was assessed. We found that in Part B subjects (one VLP dose), after 21 months of VLP vaccination, the GMT values were higher in VLP recipients than in the placebo group. Our results are in agreement with other studies, where it has been reported that antibodies to the A(H1N1)pdm09 virus can still be detected in adults up to at least 12 months after vaccination with various inactivated, split-virion H1N1 pandemic influenza vaccines [39–44, 48, 49]. Consistent with our findings, Landry et al., recently showed that a non-adjuvanted plant-made H1N1 pandemic influenza virus VLP vaccine induced detectable antibody levels up to 6 months after vaccination [12].

In addition, we also found that after 25 months, antibodies to A(H1N1)pdm09 virus were still detectable in the VLP subjects from Part A (two VLP doses). However, in subjects from this group we did not observe differences in GMT or seroprotection levels between VLP and placebo groups. Some of the reasons why we could not find differences in the antibody titres could be initially related to the sample size, and also to the fact that after 25 months, we found placebo recipients with high antibody titres. Although none of the subjects from this group reported having received seasonal influenza vaccination or presented any influenza-like illness symptoms, we cannot rule out the possibility that they might have been exposed to the pandemic virus in form of subclinical infections, since cases kept being reported in Mexico at least up until March 2012 [52].

When analysed as a function over time, the antibody titres found after 25 months in Part A subjects, were lower across all groups when compared to the values reported at day 36 after VLP vaccination in the previous study (5 μg GMT 85, 15 μg GMT 183, 45 μg GMT 209, and placebo GMT 25). In fact, the GMT values from 25 months after VLP were close to the baseline HI titres across all groups (GMT 17–26) from the precedent study [14]. Unfortunately, we do not have data from early time points that may have contributed to further clarify the kinetics of the antibody persistence induced by this VLP vaccine.

Regarding the seroprotection rate values (SPR) that were induced by vaccination, we found that 21 months after VLP vaccination, the seroprevalent and seroprotective antibody levels were higher in the VLP recipients than in the placebo subjects. Likewise, after 25 months, we found a similar tendency in both the seroprevalent and seroprotective antibody levels in VLP vaccinated subjects compared with the placebo treated subjects. The SPR values that were found in the VLP recipients were lower (22.8–29.8%), compared with those reported in other egg-produced, non-adjuvanted, pandemic H1N1 vaccines, which, in adults, reached values from 46% up to 68% after 12 months [39, 42, 49]. In the case of adjuvanted vaccines (with MF59 or AS03), the SPR values reported elsewhere ranged from 35% [43] up to above 70% [40, 44, 48]. In our study, VLP-induced SPR values were measured 21 and 25 months after vaccination, which may account for the lower levels detected compared with the above-mentioned vaccines.

Studies addressing the effect of influenza pre-vaccination over antibody responses that are induced by vaccination with inactivated vaccines have shown some conflicting data. While some studies assigned a deleterious role of previous influenza vaccinations on subsequent immunisations for seasonal, influenza B and pandemic viruses [23, 53, 54], others have reported no effects [55]. In this study, we showed that vaccination with a seasonal trivalent inactivated influenza virus vaccine after VLP vaccination increased antibody titres that reached seroprotective levels. Furthermore, the seroprotective rates in the individuals who reported receiving IIV revaccination some time after VLP immunisation were higher compared with those in the placebo recipients. This findings suggest that VLP vaccination does not have a deleterious effect on antibody responses to subsequent influenza IIV vaccinations, as we observed a boosting effect on antibody titres in IIV revaccinated VLP recipients.

The trivalent IIV vaccine used in Mexico was comprised of the A/California/07/2009 (H1N1) strain, which was closely related with the pandemic strain that was included in the VLP vaccine (A/California/04/2009/H1N1) [56]. It was previously shown that pandemic influenza vaccination can re-activate memory B-cell responses [57], which suggests that the augmented antibody responses that were observed in the VLP vaccinated subjects might be due to the re-activation of memory B cell responses. In a recent study by Eidem et al., health-care workers were first vaccinated with an AS03-adjuvanted monovalent split virion H1N1 pandemic influenza vaccine and then re-vaccinated twice with seasonal IIV. Then, the antibody titres were measured 12 months after each re-vaccination [43]. In this study, the authors found that in spite of having high antibody titres 21 days post-vaccination, the antibody persistence declined 12 months after the first immunisation, and that antibody levels increased after re-vaccination with IIV. They concluded that re-vaccination with the seasonal influenza vaccines contributed to maintain the antibody titres above seroprotective levels. Additionally, they found that revaccination also promoted affinity maturation, which resulted in superior virus neutralisation. A deeper analysis of the features of antibody responses that are elicited by revaccination with a seasonal IIV would be needed to evaluate if the same effects may occur in the VLP vaccinated subjects from our study. Nevertheless, in terms of the effect of revaccination with a seasonal trivalent inactivated vaccine after VLP vaccination, our study has a limitation: the time that passed between IIV revaccination and the first VLP immunisation was not the same across the subjects who reported receiving IIV revaccination. In order to better understand the effect on antibody responses that this may have, it would be necessary to increase the sample size and to control the revaccination timing.

The present study is the first to show the persistence of antibody responses in adults that were induced by a non-adjuvanted, monovalent, insect cell-derived 2009 H1N1 pandemic influenza virus VLP vaccine up to 25 months after vaccination. Moreover, our study is the first to show the antibody persistence that was induced by a VLP vaccine that was tested during the second wave of the 2009 pandemic in Mexico City. In conclusion, this study shows that an insect cell-derived H1N1 pandemic influenza VLP vaccine induced antibody persistence for up to two years after vaccination. Moreover, it also shows that in subjects who reported receiving revaccination with a seasonal trivalent inactivated vaccine, the presence of seroprotective antibody levels was favoured. This information could be useful for the advisory committees of influenza vaccination campaigns, especially those regarding pandemic outbreaks.

## Materials and Methods

### Ethics statement

This study was evaluated and approved by The Mexican Social Security Institute (IMSS) through the National Commission of Scientific Research (CNIC), which is composed of scientific, ethics and biosafety committees (approval number: CNIC: 2011-785-055). Written informed consent was obtained from each subject according to current good clinical practice (GCP) guidelines and to the principles expressed in the Declaration of Helsinki.

### Subjects and study design

This is a cross-sectional study from a cohort of subjects who had previously participated in a phase 2, randomised, double blind, placebo-controlled study to evaluate the safety, tolerability, and immunogenicity of an experimental and unlicensed influenza VLP vaccine that was produced by Novavax, against the pandemic A/California/04/2009 H1N1 virus (ClinicalTrials.gov Identifier: NCT01072799) [14]. VLPs were produced in *Spodofera frugiperda* Sf9 cells infected with recombinant insect baculovirus *Autographa californica* Nuclear Polyhedrosis Virus (AcNPV) expressing HA and NA genes, derived from influenza A/California/04/2009 (H1N1). Each dose contained 5 μg, 15 μg or 45μg of HA. The vehicle used was neutral phosphate buffer, which was also administered as the placebo control [14].

The preceding study was carried out in two stages (S1 Fig). Part A was performed to evaluate the safety and immunogenicity of three doses of VLP vaccine. In this part subjects were immunised with 5 μg, 15 μg or 45 μg of a haemagglutinin (HA) VLP vaccine or a placebo, with a boost on day 22. Blood samples were taken at day 1, 14, and 36. Whereas in Part B, the volunteers received a single dose of 15 μg VLP or placebo injection on day 1 and assessed for safety. No blood samples were taken from Part B subjects.

For the current study, blood samples were taken from subjects recruited at 17–29 months after they received their first VLP vaccination (i.e. July 2011-March 2012). Individual serum samples were stored at -80°C until analysis. All of the subjects provided signed informed consent prior to the start of any procedures. The medical records of each volunteer were requested, and subjects that were immunocompromised, under immunosuppressive therapy, or had uncompensated chronic diseases were not included. All of the information regarding influenza infections or influenza-like illnesses (ILI), as well as the administration of any anti-influenza vaccine as of the last VLP vaccination up until the enrolment into this study was registered.

### Haemagglutination inhibition assay

Haemagglutination inhibition (HI) assays were performed on sera that was pre-treated with receptor destroying enzyme (RDE, Denka Seiken, 370013) to remove nonspecific inhibitors, as described previously [58]. Briefly, three volumes of RDE were added to one volume of serum, and it was incubated for 18 h at 37°C and then heated at 56°C for 1 h to inactivate the residual RDE. Finally, six volumes of physiological saline (0.85% NaCl) were added to reach a final dilution of anti-sera of 1:10. The HI assay was conducted on all samples with A/Mexico/4482/2009 (H1N1) influenza virus grown in egg embryos. The HI was performed with 0.5% chicken red blood cells and serial two-fold dilutions of serum. U-bottom plates (96-well Nunc, 449824) were used to perform the assay. Before each assay, the virus titre was standardised to a dilution of 8 haemagglutination units/50 μL PBS pH 7.0.

### Statistical analysis

The sample size was based on a comparison of two proportions, and a difference of at least 20% was assumed to achieve the protective antibody titres between the groups receiving two VLP doses (Part A), versus the group that received one VLP dose (Part B). It was estimated that it was necessary to evaluate at least 173 participants per group. The sample size was calculated using the EPIDAT v. 3.1 software [59, 60]. Antibody titres are expressed in log^10, geometric mean titres (GMT) and 95% confidence intervals (95% CI). Comparisons between groups were performed using the Chi-square test, Fisher’s exact test, non-parametric Student’s t-test (Mann-Whitney U test) or non-parametric ANOVA test (Kruskal-Wallis test), as appropriate. All of the statistical analyses were performed with SPSS v.20 (IBM).

## Supporting Information

S1 Fig Previous and current study design. (A) The preceding study was carried out in two stages (López-Macías C, et al., *Vaccine*, 2011). Part A was performed to evaluate the safety and immunogenicity of three doses of VLP vaccine. In this part subjects were immunised with either 5 μg, 15 μg or 45 μg of a haemagglutinin (HA) VLP vaccine or a placebo, with a boost on day 22. Blood samples were taken at day 1, 14, and 36 post immunisation (p.i.). Whereas in Part B of the previous study, the volunteers received a single dose of 15 μg VLP or placebo injection on day 1 and assessed for safety, and no blood samples were taken. (B) For the current study, a representative sample comprising subjects from Part A and Part B from the previous study were recruited. One blood sample was taken from subjects recruited at 24 (17–29) months after they received their first VLP dose. Subjects who reported receiving inactivated influenza virus vaccine (IIV) were analysed separately. (C) Timeline of the previous and current studies with respect to the H1N1 2009 influenza season.
(TIFF)
