# Supplementary figures and images for: Antibody Persistence in Adults Two Years after Vaccination with an H1N1 2009 Pandemic Influenza Virus-Like Particle Vaccine
Source: PLoS One. 2016 Feb 26;11(2):e0150146. doi: 10.1371/journal.pone.0150146 (PMC4769292; doi:10.1371/journal.pone.0150146)

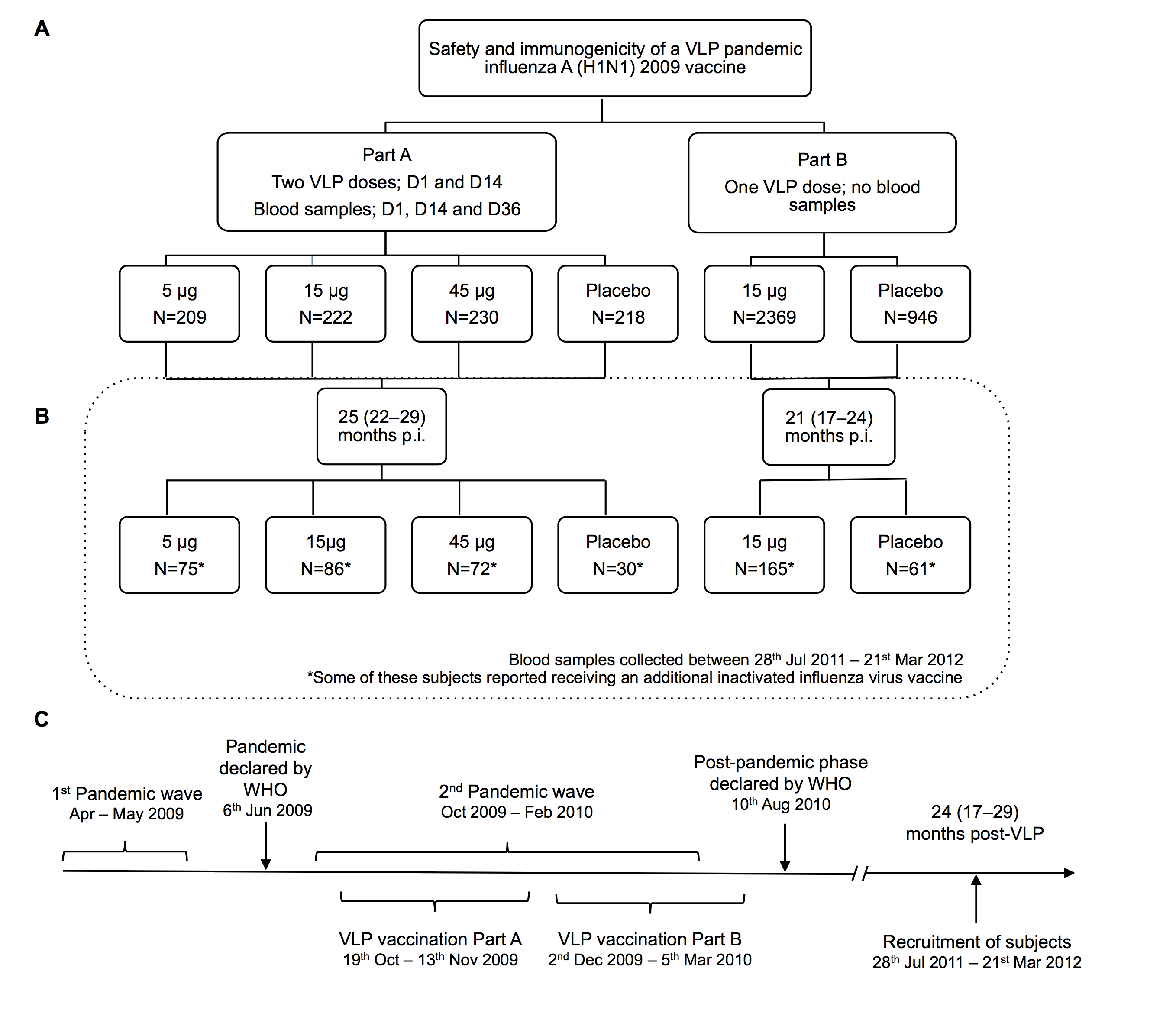

Supplement: S1 Fig — (A) The preceding study was carried out in two stages (López-Macías C, et al., Vaccine, 2011). Part A was performed to evaluate the safety and immunogenicity of three doses of VLP vaccine. In this part subjects were immunised with either 5 μg, 15 μg or 45 μg of a haemagglutinin (HA) VLP vaccine or a placebo, with a boost on day 22. Blood samples were taken at day 1, 14, and 36 post immunisation (p.i.). Whereas in Part B of the previous study, the volunteers received a single dose of 15 μg VLP or placebo injection on day 1 and assessed for safety, and no blood samples were taken. (B) For the current study, a representative sample comprising subjects from Part A and Part B from the previous study were recruited. One blood sample was taken from subjects recruited at 24 (17–29) months after they received their first VLP dose. Subjects who reported receiving inactivated influenza virus vaccine (IIV) were analysed separately. (C) Timeline of the previous and current studies with respect to the H1N1 2009 influenza season. (TIFF) [file pone.0150146.s001.tiff]
